# An origin of ultraslow spreading ridges for the Yarlung-Tsangpo ophiolites

**DOI:** 10.1016/j.fmre.2021.07.002

**Published:** 2021-07-21

**Authors:** Chuan-Zhou Liu, Fu-Yuan Wu, Tong Liu, Chang Zhang, Wei-Qi Zhang, Zhen-Yu Zhang, Zhen Zhang, Wu Wei, Yin-Zheng Lin

**Affiliations:** a State Key Laboratory of Lithospheric Evolution, Institute of Geology and Geophysics, Chinese Academy of Sciences, Beijing 100029, China; b CAS Center for Excellence in Deep Earth Science, Guangzhou 510640, China; c University of Chinese Academy of Sciences, Beijing 100049, China

**Keywords:** Yarlung-Tsangpo ophiolites, Tibetan plateau, Ultraslow spreading ridges, Subduction re-initiation

## Abstract

As relics of ancient ocean lithosphere, ophiolites are the most important petrological evidence for marking the sutures and also play a key role in reconstructing plate configuration. They also provide valuable windows for studying crustal accretion and mantle processes occurring at modern ocean ridges. Abundant ophiolites are distributed along the Yarlung-Tsangpo suture and represent the relics of ocean lithosphere of the Neo-Tethys. They are characterized by an incomplete litho-stratigraphy, of which the mantle section is much thicker than the crustal section. Ocean crustal rocks outcropped in the Yarlung-Tsangpo ophiolites are much thinner than normal ocean crusts (~ 7 km) or even absent. Tectonic settings from which the Yarlung-Tsangpo ophiolites originated remain highly controversial, although an origin of the supra-subduction zone is prevailing. Moreover, their incomplete litho-stratigraphy has been commonly attributed to tectonic dismemberment during the late-stage emplacement after their formation. Nevertheless, such an incompleteness resembles the ocean lithosphere generated at modern ultraslow spreading ridges, such as the Southwest Indian Ridge (SWIR). In this paper, we present several lines of evidence that support the formation of the Yarlung-Tsangpo ophiolites at ultraslow spreading ridges, during which detachment faults were developed. This suggests that the Yarlung-Tsangpo ophiolites might represent the ocean core complexes (OCC) in the Neo-Tethys Ocean. The OCC with high topography in the seafloor were clogged in the trench and preserved as ophiolites through Indo-Eurasia collision. The clogging resulted in the demise of an old subduction and a new subduction was re-initiated beneath the clogged OCC.

## 1 Introduction

Ophiolites have been termed as a suite of ultramafic-mafic complexes widely outcropped in global orogenic belts, which have been regarded as relics of ancient ocean lithosphere formed through seafloor spreading at ocean ridges in the plate tectonics theory. According to the definition of the Penrose Conference [1], ophiolites are constituted of two lithological sequences, i.e., the mantle sequence consisting of mantle peridotites and the crustal sequence including, from bottom to top, gabbros, sheeted dykes and pillowed basalts. An ideal ophiolite should have a crustal thickness of ~ 7 km, which has been proved to be comparable to ocean crusts generated in modern ocean ridges [2], e.g., the East Pacific Rise. At the dawn of the plate tectonic revolution, ophiolites have been suggested to form at mid-ocean ridges [3]. Nevertheless, later abundant studies have shown that ophiolites could be generated at different tectonic settings [4, 5]; in particular, most (> 90%) global ophiolites have a genetical relationship with subduction zones [6]. Tectonic settings under which ophiolites were generated remain controversial and are still a topic of debate [7].

Moreover, numerous studies have shown that only a very few of global ophiolites show similarities in litho-stratigraphy to the Penrose definition, e.g., the Oman and Troodos ophiolites. Most of global ophiolites (> 90%) have incomplete litho-stratigraphic sequences, i.e., one or several lithological components are missing; in particular, their crustal rocks have much thinner thickness compared to the Penrose-type ophiolite [3]. Their incompleteness has been commonly attributed to tectonic dismemberment during their emplacement from oceans to continents [3]. Nevertheless, it is well known that the thickness of ocean crust is highly dependent on the spreading rates of ocean ridges [8], according to which the global ocean ridges can be classified into three types, i.e., fast-, slow- and ultraslow spreading ridges [9]. Compared to fast- and slow-spreading ridges, ocean crusts generated at ultraslow spreading ridges have much thinner thickness. Without enough magma supplies, detachment faults are developed at ultraslow spreading ridges, which exhume the lower ocean crust and/or mantle peridotites at the seafloors as ocean core complexes [10, 11]. In the American Geophysical Union Chapman Conference [12], a new model has been proposed for ocean lithospheres formed at ultraslow spreading ridges through detachment faults, i.e., the Chapman model. Compared to the Penrose-type ophiolites, the Chapman-type ophiolites should be characterized by incomplete sequences of lithological units [13].

In the past decade, several studies have recognized Chapman-type ophiolites using various evidence including structural geology, paleomagnetism and petrology [13], [14], [15], [16]. Abundant ophiolitic massifs crop out along the Yarlung-Tsangpo Suture (YTS) in the Tibetan Plateau, which is commonly regarded as marking the locus of collision between the Indian and Asian plates [17], [18], [19]. The Yarlung-Tsangpo ophiolites have been extensively studied for more than fifty years [6, 18, [20], [21], [22], [23], [24], [25], [26], [27], [28], [29], [30], [31], [32], [33], [34], [35], [36], [37], [38], [39], [40], [41], [42]], but their formation settings remain highly controversial, and different models have been proposed. In recent years, discovery of ultra-high pressure and ultra-reducing minerals in both chromitites and mantle peridotites of the Yarlung-Tsangpo has stimulated a new round of studies, as they might represent the very few windows to the mantle transition zone [43]. In this study, we present petrological and geological evidence to support that the Yarlung-Tsangpo ophiolites originated at ultraslow spreading ridges.

## 2 Geological background

**Fig. 1:**
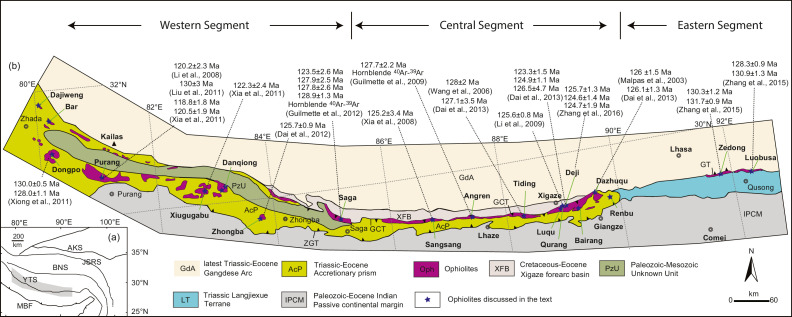
**Sketch map of the Yarlung-Tsangpo ophiolites, modified after Dai et al.**[21]. Zircon U-Pb ages of representative ophiolites are compiled in [47], and hornblende Ar-Ar ages of metamorphic soles are from [23,25].

The Tibetan Plateau is an orogenic collage composed of various terranes [44], which are separated by several well-established sutures, e.g., the Yarlung-Tsangpo Suture (YTS), the Bangong-Nujiang Suture and the Jingsha River Suture (Fig. 1(a)). The YTS is the southernmost one, which separates the Eurasia plate to the north from the India plate to the south. Ophiolitic massifs are discontinuously distributed along the YTS (Fig. 1(b)) and have been commonly regarded as relics of the Neo-Tethys Ocean, which opened during the Middle-Late Triassic [45] and closed at the end of the Cretaceous [46]. The Yarlung-Tsangpo ophiolites have been geographically divided into three segments (Fig. 1(b)), i.e., the western, central and eastern segments. Geochronological studies have suggested that the Yarlung-Tsangpo ophiolites were formed coevally at ~130–120 Ma (Fig. 1(b); and also see the compilation in [47]), although a few older ages have been also reported [20, 48, 49].

**Fig. 2:**
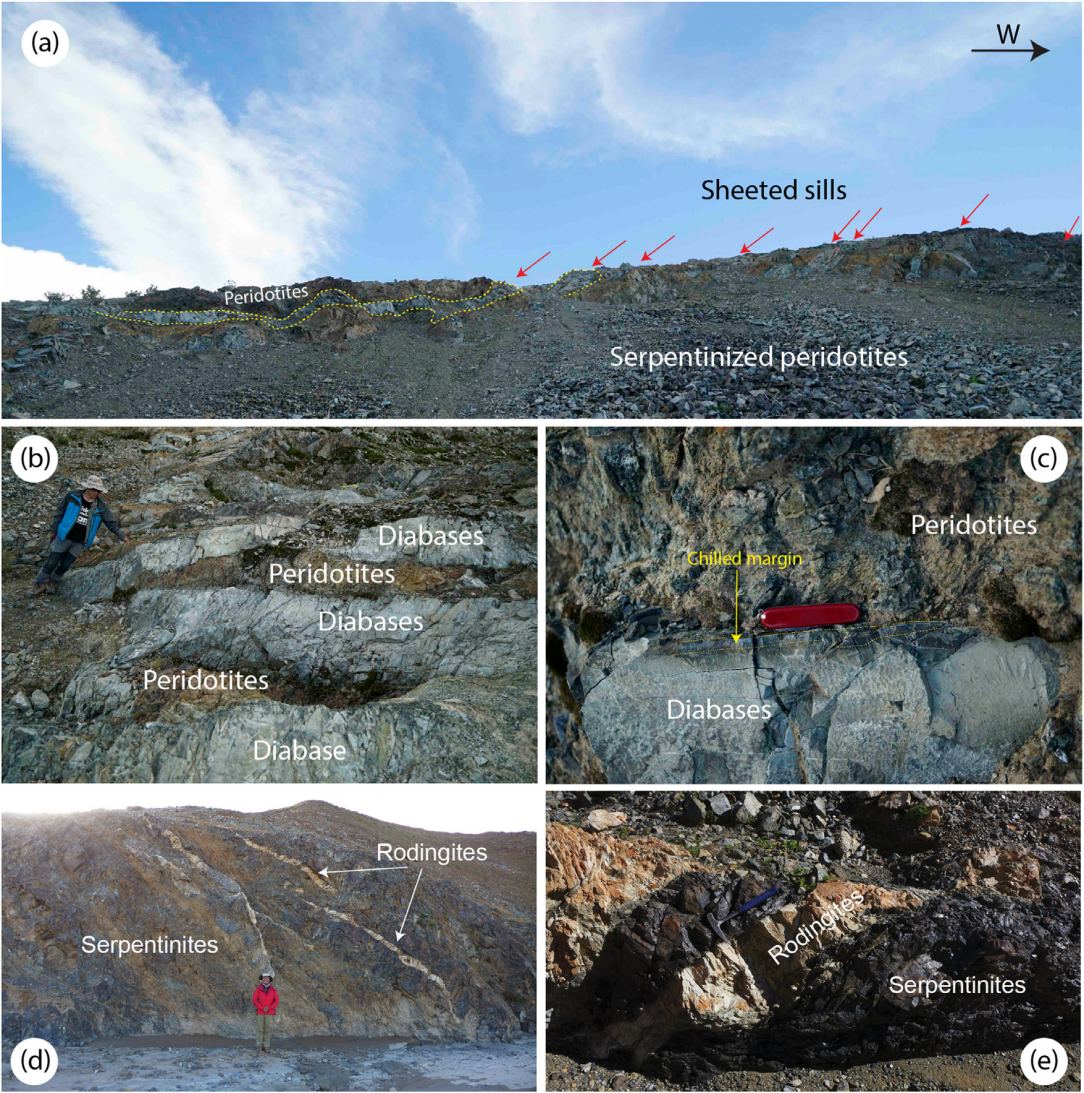
**Field photos of mafic dykes intruding mantle peridotites of the Yarlung-Tsangpo ophiolites.** (a-b) Sheeted diabase sills intruding mantle peridotites, with development of chilled margins (c). (d-e) Mafic dykes have been transformed to rodingites.

**Fig. 3:**
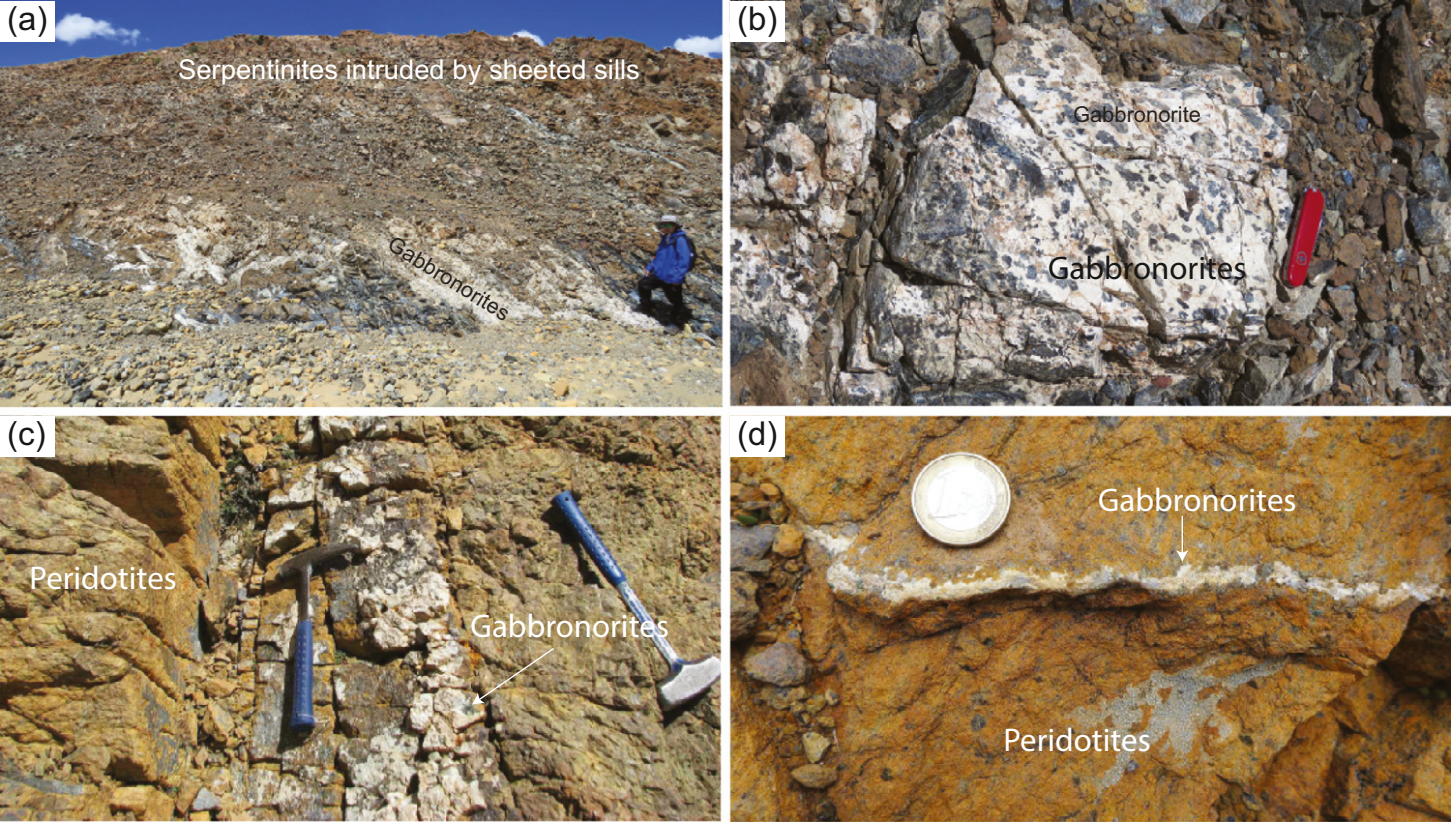
**Field photos of gabbronorites in the Yarlung-Tsangpo ophiolites.** (a-b) gabbronorite patches and (c-d) gabbronorite veins in the peridotites.

The Yarlung-Tsangpo ophiolites overall are characterized by the predominance of mantle peridotites over ocean crustal rocks, and mantle peridotites are mainly composed of refractory harzburgites, with minor lherzolites and dunites [18, 29, 33, 35, 39, 40]. Ocean crustal rocks are volumetrically small [30, 32], or even absent in some ophiolites (e.g., the Purang ophiolite; [29]). Sheeted dykes have not been discovered in any Yarlung-Tsangpo ophiolites. In contrast, diabases occur as sheeted sills intruding into both mantle peridotites and pillowed basalts [18]. In the crust-mantle transition zones, intrusions of sheeted sills into serpentinized mantle peridotites are very common in the central segment of the Yarlung-Tsangpo ophiolites, i.e., the Xigaze ophiolites (Fig. 2(a)). Chilled margins can be observed at the boundaries of these sills (Fig. 2(b, c)). On the other hand, mafic rocks also occur as dykes intruding into mantle peridotites in the lower mantle section or as blocks in the mélanges with a matrix of serpentinites (Fig. 2(d, e)). Locally, patches of pegmatitic gabbronorites can also be seen near the crust-mantle transition zones, which connect to gabbronoritic veinlets (Fig. 3(a, b)). Lower crustal gabbros are not developed in almost all Yarlung-Tsangpo ophiolites but a few, such as Jidding, Dazhuqu and Baigang [30]. Pillow basalts are widely distributed in the Yarlung-Tsangpo ophiolites, in particular in the Xigaze ophiolites, which are interbedded with radiolarian cherts [50].

The Yarlung-Tsangpo ophiolites have been extensively studied and different models have been proposed regarding their original tectonic settings [18, 21, 26, 27, 35, 42, 51, 52]. Early Sino-French studies have suggested that the Xigaze ophiolites in the central segment were generated at slow spreading ridges, as they have much thinner crustal sections compared to the Penrose-type ocean lithosphere [18, 53, 54]. Pearce and Deng [52] demonstrated that the pillow lavas in the Xigaze ophiolites show affinities to mid-ocean ridge basalts (MORB) and suggested that they were produced at the ridge transform intersection. Later studies, mainly based on geochemical compositions of both ultramafic and mafic rocks, have commonly suggested that the Yarlung-Tsangpo ophiolites were produced under subduction-related settings, either in forearc [21, 35, 42]or in back-arc [26] basins. More recently, nevertheless, several studies have proposed that the Yarlung-Tsangpo ophiolites represent the ocean core complex generated through detachment faults [27, 34, 41, 51, 55], probably at ultraslow spreading ridges [27, 34, 41].

## 3 Discussions

### 3.1 Ultra-refractory mantle domains in the Yarlung-Tsangpo ophiolites

**Fig. 4:**
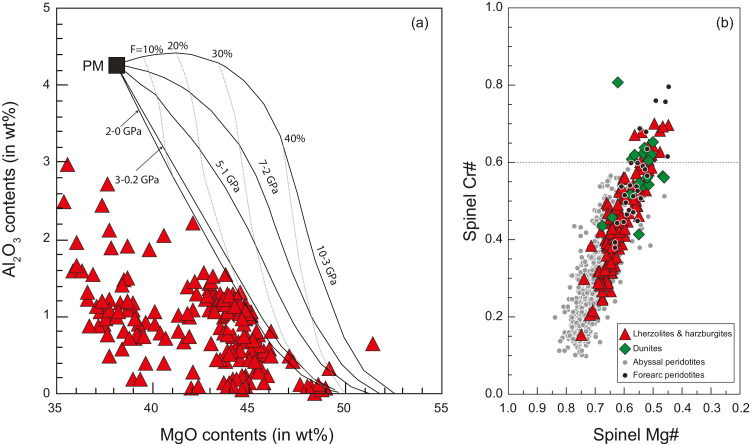
**Major elements of mantle peridotites from the Yarlung-Tsangpo ophiolites.** (a) Whole rock MgO vs Al_2_O_3_ contents; (b) Spinel Mg# vs Cr# values. Data of the Yarlung-Tsangpo mantle peridotites are from [20,27,28,31,33,[35], [36], [37], [38], [39],^42^,^58^,^95^], abyssal peridotites are from [58], and forearc peridotites are from [59].

Abundant studies have revealed that refractory harzburgites are predominated over relatively fertile lherzolites in mantle sections of the Yarlung-Tsangpo ophiolites [18, 29, 33, 35, 39, 40]. The harzburgites are characterized by high whole rock MgO contents but low contents of both Al_2_O_3_ and CaO, indicating they are mantle residues after high degrees of partial melting (Fig. 4(a)). Moreover, the spinel Cr# value has also been regarded as a proxy for melt depletion of mantle peridotites, which increases along with the degree of partial melting [56, 57]. The Yarlung-Tsangpo mantle peridotites have a large variation in spinel Cr# (Fig. 4(b)); in particular, mantle peridotites from several ophiolitic massifs (e.g., Luqu and Luobusa) have spinel Cr# higher than 0.6, which has been suggested as the upper limit for abyssal peridotites experienced anhydrous melting beneath mid-ocean ridges [56], [57], [58]. Harzburgites with spinel Cr# values > 0.6 can result from hydrous melting of mantle peridotites and thus have been commonly interpreted to originate from a forearc setting [59]. This has led to a supra-subduction zone (SSZ) origin for the Yarlung-Tsangpo ophiolites [20]. Nevertheless, refractory harzburgites with similarly high spinel Cr# (i.e., >0.6) have been also discovered at modern ocean ridges (e.g., the Mid-Atlantic Ridge), which might represent the recycled mantle wedge peridotites [60]. Moreover, abyssal peridotites with compositions as refractory as cratonic mantle peridotites have also been discovered in the Southwest Indian Ridge (Liu CZ, unpublished data). Therefore, fertility of mantle peridotites cannot be arbitrarily applied to discriminate their tectonic settings.

**Fig. 5:**
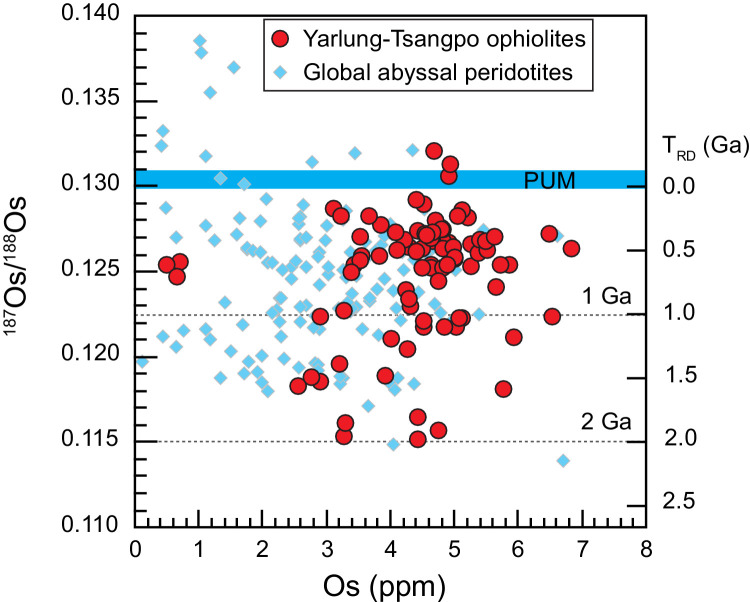
**Whole rock Os isotopes of mantle peridotites from the Yarlung-Tsangpo ophiolites.** Data of the Yarlung-Tsangpo mantle peridotites are from [28,31,37,38,61] and abyssal peridotites are from [62], [63], [64], [65], [66].

Ages of the Yarlung-Tsangpo mantle peridotites have been constrained using *Re*-Os isotopes (Fig. 5). Although most Yarlung-Tsangpo mantle peridotites have relatively juvenile ages less than 1 Ga, some refractory harzburgites from different ophiolitic massifs have highly unradiogenic ^187^Os/^188^Os ratios, yielding old Os model ages up to 2 Ga [28, 31, 37, 38, 61]. This suggests that some mantle domains within the Yarlung-Tsangpo ophiolites have experienced ancient partial melting that was much older than spreading-related magmatism at the Neo-Tethys Ocean [47]. They should represent ancient refractory mantle domains within the asthenosphere, as inferred by studies on modern abyssal peridotites [62], [63], [64], [65], [66]. Therefore, some portions of the Yarlung-Tsangpo mantle peridotites represent recycled mantle within the asthenosphere [28, 31, 37, 38], which might act as the hosting ‘tanker’ for the ultra-high pressure and ultra-reducing minerals recently discovered [43].

### 3.2 Low magma supply during formation of the Yarlung-Tsangpo ophiolites

Chemical compositions of mantle peridotites in the Yarlung-Tsangpo ophiolites suggest that they have experienced relatively high degrees of partial melting. Therefore, thick ocean crusts are expected for the Yarlung-Tsangpo ophiolites. Nevertheless, ocean crusts in all the Yarlung-Tsangpo ophiolites are much thinner than the normal ocean crusts with a thickness of ~ 7 km [18, 67]. In particular, several ophiolitic massifs (e.g., the Purang ophiolite) do not contain any ocean crustal rocks at all and only mantle peridotites are outcropped [27], [28], [29]. Plutonic gabbros on a scale of kilometers have only discovered in a few ophiolitic massifs (i.e., Jiding, Dazhuqu and Baigang) at the central segment of the Yarlung-Tsangpo ophiolites [30].

**Fig. 6:**
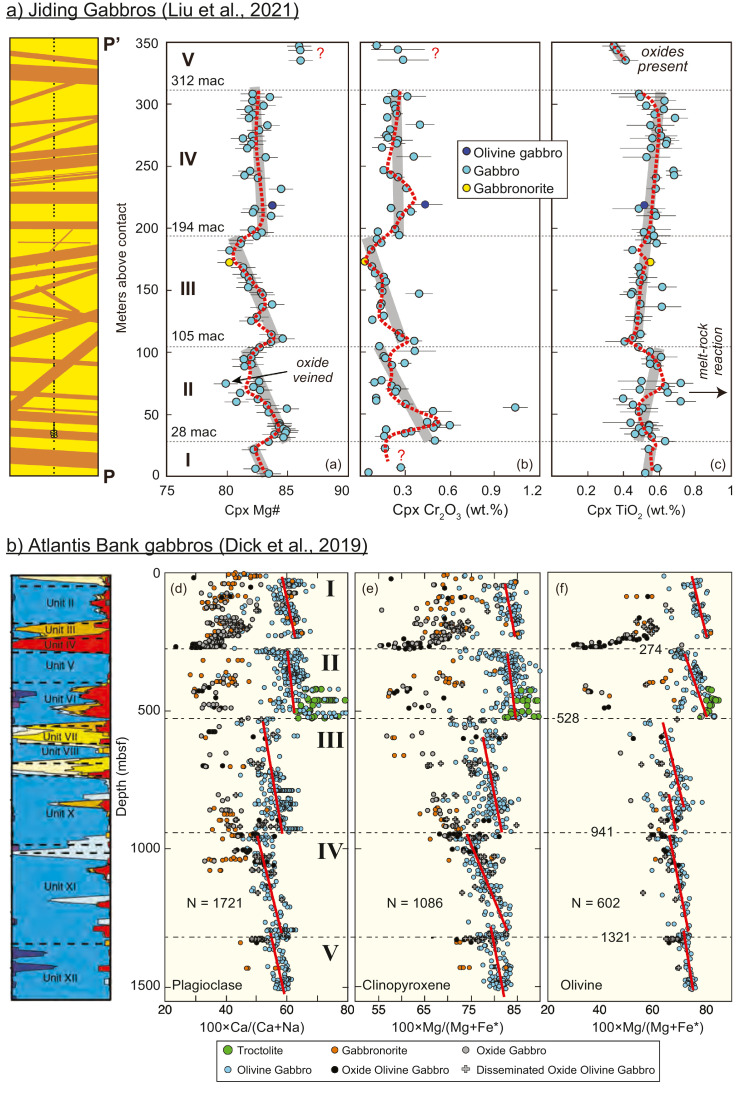
**Chemical variations of gabbros from the Jiding ophiolite****[30]****(a) and the Atlantis Bank, Southwest Indian Ridge****[72]****(b).**

In the Jiding massif, plutonic gabbros representing the lower ocean crust with a thickness of ~ 350 m are exposed, which are intruded by numerous diabase dykes [30]. Their microtextures clearly show that plagioclase is in a euhedral to subhedral shape and crystallized earlier than clinopyroxene. Such a crystallization order is consistent with low-pressure crystallization of dry basaltic magmas [68], but distinctly different from fractionation of subduction-related basalts, of which high water contents result in earlier crystallization of clinopyroxene than plagioclase [69,70]. This implies a mid-ocean ridge origin of the Xigaze ophiolites, although anhydrous magmas (e.g., forearc basalts) could also occur in the forearc during subduction initiation [71]. A detailed mapping and systematic sampling of the Jiding gabbro section have revealed cyclic chemical variations (Fig. 6(a)), which highly resemble the gabbroic ocean core complexes at modern ultraslow-spreading ridges, e.g., the Atlantis Bank at the SWIR (Fig. 6(b); [72]). Plagioclase in the Jiding gabbros are almost completely altered and thus rarely record petrogenetic information. Based on clinopyroxene compositions (Fig. 6(a)), the Jiding gabbro section exhibits episodic chemical variations and clearly breaks down-section [30]. Such features can be explained as multiple magmatic intrusions, with upward differentiation from more primitive to more evolved liquids in each sub-unit (Fig. 6). A similar explanation has previously been proposed to account for the mineral stratigraphy of Hole 735B gabbros representing the upper 1508 m of the gabbro massif exhumed at the Atlantis Bank Ocean Core Complex (OCC), Southwest Indian Ridge [72]. Such cyclic variations in compositions of gabbros reflect low magma supplies at ultraslow spreading ridges and the controlling of detachment faults on the evolution of magmas [72, 73].

### 3.3 Hydrous melting and hydrated magmas

Although the plutonic gabbros in the Yarlung-Tsangpo ophiolites were derived from anhydrous basalts, occurrence of gabbronorites points to relatively high contents of water in their parental magmas. Gabbronorites have been widely reported in ophiolites, e.g., Oman [74], [75], [76], [77], [78], Troodos [79], Bay of Islands [80] and the Alpine–Apennine ophiolites [81], occurring either as cumulates in plutonic sequences or as dykes intruding into the mantle. They are characterized by the early crystallization of pyroxenes, in particular orthopyroxene, relative to plagioclase. Plagioclases commonly have extremely high anorthite contents (An) and pyroxenes have very high Mg# and Cr abundances, which are coupled with low Na and Ti in high-Ca pyroxenes. Such features cannot be explained easily by fractional crystallization of melts with compositions of MORB. It has been suggested that they are cumulative products of melts more water- and silica-rich than MORB [74], [75], [76], [77], [78]. Such water- and silica-rich melts have been commonly inferred to originate in subduction zones.

**Fig. 7:**
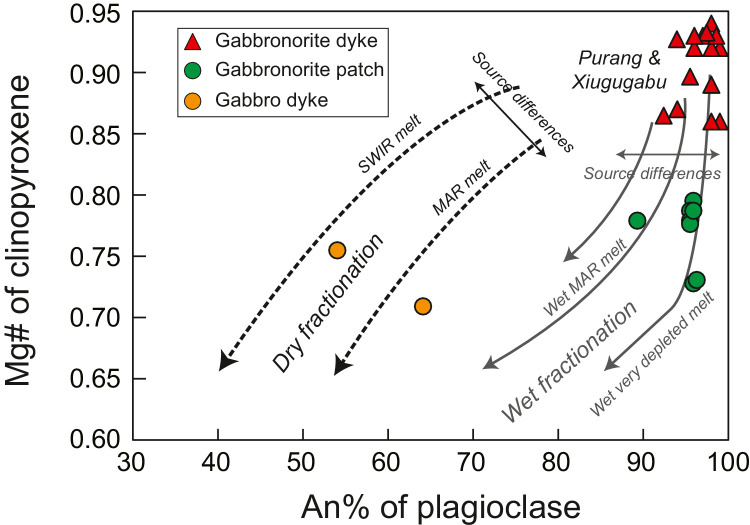
**Clinopyroxene Mg# vs plagioclase An of gabbronorites and gabbros in the Yarlung-Tsangpo ophiolites.** The fractionation trajectories are from [96]. Data of the Purang gabbronorite dykes are from [27]. Data of gabbronorite patches and gabbro dykes are unpublished.

Two types of gabbronorites crop out in the Yarlung-Tsangpo ophiolites; they occur either as coarse-grained dykes intruding fresh mantle peridotites (Fig. 3(c, d)) or as pegmatitic patches within heavily serpentinized peridotites near the crust-mantle transition zones (Fig. 3(a, b)). Our previous studies on the gabbronorite dykes in both Purang and Xiugugabu ophiolites have revealed that they have remarkably high clinopyroxene Mg# and plagioclase An contents (Fig. 7). We have proposed that they were derived from hydrous basalts through partial melting of serpentinized mantle peridotites, as a result of seawater infiltration through the detachment faults [27]. Therefore, an origin of ocean core complex at an ultraslow spreading ridge has been inferred for the Purang ophiolite [27]. Compared to the gabbronorite dykes, the patched gabbronorites have lower Mg# values of both orthopyroxene and clinopyroxene, but similar plagioclase An contents (Fig. 7). As they follow the wet fractionation trends (Fig. 7), their parental magmas should have high water contents, which is also consistent with their pegmatitic texture. A likely scenario is that the anhydrous basalts were extracted from the asthenosphere and migrated upwards. Along the migration, some basalts crystallized and gave rise to gabbros following the dry fractionation trajectory (Fig. 7). Some of them were transported to the crust-mantle transition zone, where they achieved exotic waters through assimilation of the serpentinized mantle or addition of hydrous fluids circulation along the detachment faults [82, 83]. Therefore, both types of gabbronorites bear the evidence for the development of detachment faults in the Yarlung-Tsangpo ophiolites.

### 3.4 Rapid exhumation of mantle by a detachment fault

Other than gabbros and gabbronorites, diabase is another type of mafic dykes extensively intruding into mantle peridotites (Fig. 2). At the crust-mantle transition zone, the diabase dykes occur as sheeted sills intruding into the serpentinized peridotites, with consistent strikes (Fig. 2(a, b)). They are very fresh in the interiors and display chilled margins (Fig. 2(c)). This suggests that the mantle peridotites have been already altered and thus cooled to low temperature before the intrusion of the diabase sills. Moreover, diabase dykes occur as singular intrusions in the lower part of the mantle section (Fig. 2(d, e)) and have been almost completely transformed to rodingites [84, 85]. Rodingite is a calcsilicate rock characterized by hydrogarnet, grossular, diopside and prehnite; minerals such as vesuvianite, titanite, chlorite and zeolite may also be present. Its formation has been explained by Ca-metasomatism of rocks with mafic compositions through reacting with Ca-rich fluids released during serpentinization of mantle peridotites [86]. This indicates that the mantle peridotites were still fresh when the mafic dykes, i.e., protoliths of the rodingites, intruded. Therefore, the rodingites and diabase sills are mafic intrusions before and after serpentinization of mantle peridotites, respectively.

**Fig. 8:**
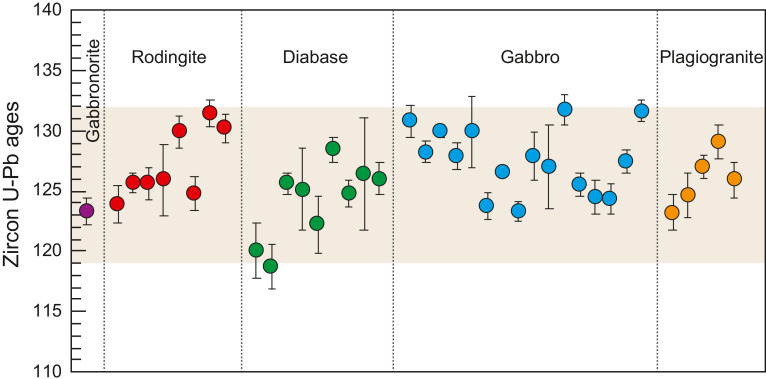
**Zircon U-Pb ages of ocean crustal rocks of the Yarlung-Tsangpo ophiolites.** Data are compiled in [47].

Abundant zircon U-Pb dating have been conducted for ocean crustal rocks (including gabbros and plagiogranites) and the mafic intrusions in the Yarlung-Tsangpo ophiolites (Fig. 8). The results revealed that both rodingites and diabase sills have identical zircon U-Pb ages within analytical uncertainties, which are also identical to the ocean crustal rocks [34, 84]. Such a geochronological simultaneity supports that the mantle peridotites were exhumed very rapidly from the deep mantle to the shallow seafloor. During the exhumation, mantle peridotites were serpentinized by the circulating seawater or hydrothermal fluids, which were accompanied by intrusions of mafic magmas [34]. The synchronism between rapid exhumation of the mantle and magmatism can only be achieved through the development of detachment faults, which are commonly developed at ultraslow spreading ridges.

### 3.5 Ocean core complex and subduction re-initiation

**Fig. 9:**
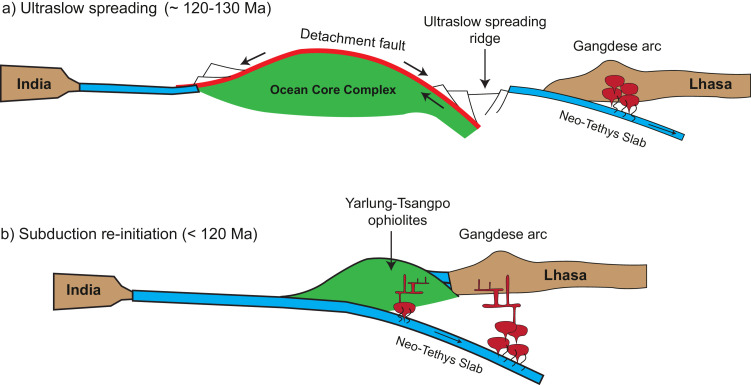
**Cartoon for the subduction re-initiation model.** (a) At ~ 130 Ma, ocean ridges in the Neo-Tethys Ocean were proximal to the Eurasian continental margins and have been slowed down to an ultraslow spreading rate. Detachment faults were developed at the ocean ridges, which resulted in the exhumation of mantle peridotites and occasionally the gabbros at the seafloor as ocean core complexes (OCC). (b) The ocean core complexes, with incomplete ocean lithospheres, were transported into the trench due to the northward subduction. They cannot be subducted due to their higher topography relative to the ambient ocean crust, and thus clogged in the trench. The clogging led to the demise of the old subduction to the north and a new subduction was re-initiated in the south of the ocean core complexes. A metamorphic sole with high temperature metamorphism was produced beneath at the bottom of the mantle section. Meanwhile, mantle peridotites of the clogged ocean core complexes might have been modified by fluids or melts released from the slabs, giving rise to the SSZ-like geochemical features.

As discussed above, different lines of evidence for mafic rocks in the Yarlung-Tsangpo ophiolites, including structures, compositions and geochronology, suggest that they were produced through detachment faults at ultraslow spreading ridges [27, 51, 55]. Therefore, the Yarlung-Tsangpo ophiolites represent relics of ocean core complexes in the Neo-Tethys Ocean [27]. Previous paleomagnetism studies have constrained the paleo-latitudes of the Yarlung-Tsangpo ophiolites, which were very close to the southern margin of the Lhasa terrane [87]. This implies that the ocean ridges, at which the Yarlung-Tsangpo ophiolites were generated, were also proximal to the trench to the north side (Fig. 9(a)). A remarkable feature of modern ocean core complexes is that they are topographically high relative to the ambient ocean crust, with a difference in elevation up to 4 km [10,88]. In particular, recovery of oolitic limestones from the Atlantis Bank ocean core complex suggests that it occurred as an island with a 1200 m peak above sea level [72]. Once entering the trench, the elevated ocean core complexes cannot be subducted and thus clogged in the trench, which were preserved and emplaced as the Yarlung-Tsangpo ophiolites during the Indo-Eurasian collision.

Clogging of an ocean core complex in the trench would result in the demise of an old subduction on one hand. On the other hand, the far-field compressional strength caused by the northward drifting of the Indian continent will initiate a new subduction in the south of the clogged ocean core complex. This is the *so-called* “subduction re-initiation” at the ultraslow spreading ridges [41]. Numerical modeling studies have demonstrated that detachment faults can act as weak zones during compression, which is conducive to subduction initiating [89, 90]. The subduction re-initiation processes were witnessed by the metamorphic soles occurring within the mélange at the bottom of the ophiolites, which consist of a suite of metamorphic rocks from greenschists, amphibolites to granulite-facies garnet clinopyroxenites [[23], [24], [25],^41^]. They were transformed from mafic protoliths with compositions similar to nascent ocean crust after metamorphism at relatively high geothermal gradients (25–30 °C/km; [41]). Subduction re-initiation due to clogging of ocean core complex is, mechanically, compression-induced, which is similar to subduction zone transference [91]. A corollary of starting a new subduction beneath the clogged ocean core complex is the modification of the mantle peridotites by the slab-released fluids/melts, which were recorded by geochemical compositions of mantle peridotites in the Yarlung-Tsangpo ophiolites [92]. In addition, subduction-related magmatism could also be superimposed on the original ridge-type rocks (Fig. 9(b)). In this sense, both SSZ- and MOR-type geochemical features are not exclusive in the subduction re-initiation model, which might also provide a potential solution to the ophiolite conundrum [93].

## 4 Conclusion

Ophiolite is an important term in Earth sciences and has a research history for more than 200 years, since first defined by the French mineralogist Alexandre Brongniart in 1813. The ophiolite concept went through several phases of evolution, among which the Penrose Conference was a milestone and the Penrose definition has been far-reaching in the ophiolite community [1]. Since then, it has become a consensus that the overwhelming majority of global ophiolites have litho-stratigraphic structures unlike the Penrose definition. Such a structural incompleteness has been attributed to tectonic dismemberment. A few ophiolites (i.e., Oman and Troodos) consistent with the Penrose definition have been regarded as the stereotype of ophiolites. Nevertheless, the orthodoxy of the Penrose definition has been strongly challenged by the research progress in the modern ocean lithosphere. In particular, studies on the ultraslow spreading ridges (with a full spreading rate < 20 mm/yr) have shown that ocean lithospheres generated by detachment faults have incomplete structures [88]. The American Geophysical Union Chapman Conference on ‘Detachments in Oceanic Lithosphere’ was held in May 2010 [12, 94], which should hallmark the paradigm shift in ophiolite studies. Our group studies on the Yarlung-Tsangpo ophiolites have proved that they are Chapman-type ophiolites and their litho-stratigraphical incompleteness is a genuine characteristic during formation rather than resulting from the late-stage tectonic dismemberment. In this sense, the Yarlung-Tsangpo ophiolites, in particular the Xigaze ophiolites, are ideal objects for studying magmatic accretion and seafloor spreading at ultraslow spreading ridges, and thus, their significance should be on a par with the Oman ophiolites [67].

## Declaration of Competing Interest

The authors declare no competing interests.
